# Supplementary material for: Early Identification of Mild Cognitive Impairment in Person with Cancer Undergoing Chemotherapy: Associations with Anxiety, Sleep Disturbance and Depression
Source: Healthcare (Basel). 2025 Nov 11;13(22):2868. doi: 10.3390/healthcare13222868 (PMC12652556; doi:10.3390/healthcare13222868)
Supplement: Supplementary file 1 [file healthcare-13-02868-s001.zip › S1_Approval of the ethics committee.pdf]

COMPLEJO ASISTENCIAL UNIVERSITARIO DE SALAMANCA
Paseo de San Vicente, 58-182
37007 Salamanca
Comité Ético de Investigación con Medicamentos
Teléfono: 923 29 11 00 – Ext. 55 515

CEIm Área de Salud de Salamanca

E-mail: comite.etico.husa@saludcastillayleon.es

Sacyl

DICTAMEN DEL COMITE DE ETICA DE LA INVESTIGACION CON MEDICAMENTOS

Doña CONCEPCIÓN TURRIÓN GÓMEZ, Secretaria Técnica del Comité de Ética de la Investigación con medicamentos del Área de Salud de Salamanca,

C E R T I F I C A

Que este Comité, en su reunión del 22/01/2024 CEIm Ref. 2024/01
ha evaluado el Proyecto de Investigación titulado

"CHEMOBRAIN": ESTUDIO DESCRIPTIVO DEL DETERIORO COGNITIVO QUIMIOINDUCIDO EN PACIENTES ONCOLÓGICOS

Código CEIm: PI **2023 12 1474 - TFM**

del que es Investigador Principal Dña Joana Rivas García

del Servicio de Oncología

valorado de acuerdo con la Ley 14/2007 de Investigación Biomédica, Principios éticos de la Declaración de Helsinki de la Asociación Médica Mundial sobre principios éticos para investigaciones médicas con seres humanos, así como el resto de principios éticos y normativa legal aplicable en función de las características del estudio,

Considera que dicho estudio cumple los requisitos necesarios y es viable para su realización en este centro, por lo que **INFORMA FAVORABLEMENTE** para la realización de dicho estudio

Y para que conste, lo firma en Salamanca con fecha 25 de enero de 2024

TURRION GOMEZ MARIA DE LA CONCEPCION - 07986126C

Firmado digitalmente por TURRION GOMEZ MARIA DE LA CONCEPCION - 07986126C

LA SECRETARIA

Fdo.: Dña. Concepción Turrión Gómez

Composición del CEIm del Área de Salud de Salamanca
Presidente: D. Luis Muñoz Bellvís (Jefe de Servicio de Cirugía General y Aparato Digestivo)
Vicepresidente: D. Enrique Nieto Manibardo (Delegado de protección de datos del CAUSA)
Secretaria: Dña. Concepción Turrión Gómez (Farmacéutica y Bioquímica - Representante Comité Científico - IBSAL).
Vocales: D. Ricardo Tostado Menéndez (Farmacólogo Clínico); Dña. Silvia Jiménez Cabrera (Farmacia Hospitalaria); Dña. Ascensión Hernández Encinas (Presidenta ASCOL, representante de los pacientes); Dña. Mª Teresa Arias Martín (Enfermera de Salud Mental. Miembro del Comité de Bioética Asistencial); Dña. Mª del Carmen Arias de la Fuente (Técnico Gestor de Ensayos Clínicos); Dña. Berta Bote Bonaechea (Especialista en Psiquiatría); Dña. Ángela Rodríguez Rodríguez (Jefa Unidad de Hematología); D. Guzmán Franch Arcas (Especialista en Cirugía General y Aparato Digestivo); D. Antonio Márquez Vera (Fisioterapeuta); Dña. Ana Martín García (Especialista en Cardiología); Dña. Teresa Martín Gómez (Especialista en Oncología); Dña. Concepción Rodríguez Barrueco (Farmacéutica de Atención Primaria); D. Manuel Ángel Gómez Marcos (Médico de Atención Primaria. Responsable de la Unidad de Investigación de Atención Primaria de Salamanca), Dña. Belén Vidriales Vicente (Jefa de Sección.Hematología)
