# Supplementary material for: Early Identification of Mild Cognitive Impairment in Person with Cancer Undergoing Chemotherapy: Associations with Anxiety, Sleep Disturbance and Depression
Source: Healthcare (Basel). 2025 Nov 11;13(22):2868. doi: 10.3390/healthcare13222868 (PMC12652556; doi:10.3390/healthcare13222868)
Supplement: Supplementary file 1 [file healthcare-13-02868-s001.zip › S2_STROBE Statementí¬Checklist.pdf]

STROBE Statement—Checklist

	Item No	Recommendation
**Title and abstract**	1	(*a*) Early Identification of Mild Cognitive Impairment in Cancer Patients Undergoing Chemotherapy: Associations with Anxiety, Sleep Disturbance and Depression
		(*b*) Provide in the abstract an informative and balanced summary of what was done and what was found
**Introduction**		
Background/rationale	2	Cancer-related cognitive impairment (CRCI) is a prevalent and underdiagnosed complication in cancer patients, influenced by treatment, aging, and psychological comorbidities, making early detection essential for preserving quality of life.
Objectives	3	This study aimed to examine the associations between cognitive performance and variables such as anxiety, sleep disturbances, and depression in cancer patients undergoing chemotherapy, hypothesizing significant correlations among these factors.
**Methods**		
Study design	4	This study aimed to examine the associations between cognitive performance and variables such as anxiety, sleep disturbances, and depression in cancer patients undergoing chemotherapy, hypothesizing significant correlations among these factors.
Setting	5	Data were collected between January 2023 and February 2025 at the Complejo Asistencial Universitario de Salamanca (Spain), in both inpatient and outpatient oncology units.
Participants	6	(*a*) 275 adult cancer patients undergoing active chemotherapy were recruited by convenience sampling, meeting defined inclusion/exclusion criteria to ensure sample homogeneity.
Variables	7	Outcomes included cognitive function, memory failures, anxiety, depression, and sleep quality; age and education were potential confounders; CRCI was operationalized using standardized diagnostic criteria.
Data sources/ measurement	8*	Validated instruments (RECF, MFE-30, HADS, PSQI) were used for assessment; all were administered in-person under standardized conditions.
Bias	9	Efforts to minimize bias included strict inclusion/exclusion criteria, validated tools, and standardized data collection by trained personnel.
Study size	10	Sample size was determined via power analysis, based on local incidence and prior studies, with an initial estimate of 184 participants expanded to 275 to increase validity
Quantitative variables	11	Quantitative variables were analyzed according to distribution; grouping by age and severity levels was applied for subgroup comparisons.
Statistical methods	12	(*a*) Pearson correlations, t-tests, and Chi-square tests were applied to explore associations; confounding was partially addressed through eligibility criteria
		(*b*) Non-probabilistic sampling limited the use of advanced sampling-based inference methods.
		(*c*) Cases with missing data were excluded from specific analyses; no imputation methods were applied.
		(*d*) Non-probabilistic sampling limited the use of advanced sampling-based inference methods.
		(*e*) Sensitivity analyses were not conducted; limitations were acknowledged and discussed in interpretation of results.

**Results**		
Participants	13*	(a) A total of 275 cancer patients were included in the final sample. These participants met all inclusion criteria, including having already initiated chemotherapy at the time of assessment.
		(b) Participants were excluded if they had not yet started chemotherapy, had non-active cancer, were in palliative care, or had a clinically recognized cognitive impairment in their medical history.
		(c) Although not included in the manuscript, a flow diagram could be added to visually represent the selection process.
Descriptive data	14*	(a) The final sample consisted of 275 cancer patients aged 20–92 years (M = 63.18; SD = 13.16); 44.4% men, 55.6% women. Education levels: 45.5% primary, 28.8% secondary, 24.4% higher education, 1.1% no schooling. Most common diagnoses: breast cancer (30%), digestive (17.7%), lung (16.6%), hematological (11.1%), others (24.4%). 83.3% received chemotherapy alone, 16.7% chemotherapy plus hormone therapy. Potential confounders considered included age, emotional distress (HADS), and sleep quality (PSQI).
		(b) The manuscript does not report missing data for any variable
Outcome data	15*	Summary measures are reported: ERFC mean score = 40.56 (SD = 12.37); MFE-30 = 15.57 (SD = 11.67); HADS = 10.07 (SD = 6.07); PSQI = 8.08 (SD = 4.52).
Main results	16	(*a*) The results present Spearman correlation coefficients: HADS and ERFC ($\rho = -0.146$; $p < 0.05$); PSQI and ERFC ($\rho = -0.583$; $p < 0.001$); age and ERFC ($\rho = -0.016$); age and PSQI ($\rho = 0.583$); HADS and age ($\rho = 0.572$). No multivariable adjustment or confidence intervals were reported.
		(*b*) Variables were analyzed as continuous. No categorization or cutoffs were applied.
		(*c*) N/A
Other analyses	17	N/A
**Discussion**		
Key results	18	The study found that poorer cognitive performance was significantly associated with emotional distress, poor sleep quality, and older age. No significant relationship was found between anxiety/depression and sleep quality. Sex and treatment lines were not significantly associated with outcomes.
Limitations	19	Limitations discussed include the cross-sectional design (no causality), single-center sample, and the lack of full neuropsychological evaluation or neurobiological markers. These may limit generalizability and depth of mechanistic understanding.
Interpretation	20	The study supports the multifactorial nature of CRCI and highlights the influence of age, sleep, and emotional well-being. Despite limitations, results are aligned with previous literature and emphasize the need for early screening and individualized interventions in oncology
Generalisability	21	The study's generalizability may be limited by its single-institution setting. However, the broad age range and inclusion of both sexes and multiple cancer types enhance clinical relevance
**Other information**		
Funding	22	This research received no external funding
